# Mixed Receptive Fields Augmented YOLO with Multi-Path Spatial Pyramid Pooling for Steel Surface Defect Detection

**DOI:** 10.3390/s23115114

**Published:** 2023-05-27

**Authors:** Kewen Xia, Zhongliang Lv, Chuande Zhou, Guojun Gu, Zhiqiang Zhao, Kang Liu, Zelun Li

**Affiliations:** College of Mechanical and Power Engineering, Chongqing University of Science and Technology, Chongqing 401331, China; 2021203258@cqust.edu.cn (K.X.); 2021203218@cqust.edu.cn (G.G.); 2021203266@cqust.edu.cn (Z.Z.); 2022203026@cqust.edu.cn (K.L.); 2008020@cqust.edu.cn (Z.L.)

**Keywords:** mixed receptive fields, multi-path spatial pyramid pooling, re-parameterized conv, steel surface defect detection, YOLOv5s

## Abstract

Aiming at the problems of low detection efficiency and poor detection accuracy caused by texture feature interference and dramatic changes in the scale of defect on steel surfaces, an improved YOLOv5s model is proposed. In this study, we propose a novel re-parameterized large kernel C3 module, which enables the model to obtain a larger effective receptive field and improve the ability of feature extraction under complex texture interference. Moreover, we construct a feature fusion structure with a multi-path spatial pyramid pooling module to adapt to the scale variation of steel surface defects. Finally, we propose a training strategy that applies different kernel sizes for feature maps of different scales so that the receptive field of the model can adapt to the scale changes of the feature maps to the greatest extent. The experiment on the NEU-DET dataset shows that our model improved the detection accuracy of crazing and rolled in-scale, which contain a large number of weak texture features and are densely distributed by 14.4% and 11.1%, respectively. Additionally, the detection accuracy of inclusion and scratched defects with prominent scale changes and significant shape features was improved by 10.5% and 6.6%, respectively. Meanwhile, the mean average precision value reaches 76.8%, compared with the YOLOv5s and YOLOv8s, which increased by 8.6% and 3.7%, respectively.

## 1. Introduction

During the production process of steel, various types of defects, such as cracks, holes, and scratches, may form on the surface of the product due to factors, such as raw material quality, manufacturing equipment, and production environment [1]. Among the typical defects on steel surfaces, different types of defects usually differ greatly in shape, size, and distribution due to various factors, which is challenging for the design of detectors [2]. In addition, with the rapid development of high-precision machinery manufacturing, higher quality requirements are put forward for the surface process quality inspection of steel products, especially the detection of subtle defects on the steel surface [3]. In the current research of steel surface defect detection methods, traditional image-based detection methods and machine learning-based detection methods rely on features, such as texture, edge contours, and contrast, to identify defects and require complex feature extraction algorithms and feature classifiers for complex designs, and there are still some shortcomings in the face of the above detection challenges [4]. Deep learning-based detection methods can achieve certain effects in addressing the above detection challenges through targeted algorithms, such as data augmentation [5], multi-scale feature fusion [6], and attention mechanisms [7,8]. However, they still have a certain distance from high-precision detection of steel surfaces [9]. Therefore, it is of great research significance to develop a high-precision steel surface defect detection algorithm that can cope with steel surface defect scale changes, background texture interference, and subtle defects.

In the research of steel surface defect detection based on traditional image-based detection methods. Wen et al. [10] proposed a detection algorithm that combines grayscale images and three-dimensional depth information. This algorithm combines the rough compactness measurement of regions of interest with depth information and has a good detection ability for defects on the surface of steel with unevenness. Xu et al. [11] proposed a detection algorithm that introduces Shearlet transform to provide efficient multi-scale directional representation, which achieved good results in testing on different steel production lines. Yan et al. [12] proposed an image preprocessing algorithm that combines improved principal component analysis with a genetic algorithm for feature selection. This algorithm can intelligently and quickly screen out suspected defect images on the surface of round steel by using evolutionary calculation and parallel calculation based on compute unified device architecture. Although traditional detection algorithms based on image processing can bring certain detection effects, these methods usually require strict application conditions, such as high stability in lighting, posture, and texture. In terms of robustness and real-time performance, these algorithms cannot better meet industrial needs.

In the research of steel surface defect detection based on machine learning methods. Chu et al. [13] proposed a multi-information twin support vector machine aiming at the efficiency and accuracy of steel surface defect classification. The algorithm has good execution efficiency and anti-noise performance. Yue et al. [14] proposed an improved bat algorithm, which enables the optimized backpropagation network to have higher accuracy in steel defect detection. Wang et al. [15] proposed an improved random forest algorithm with optimal multi-feature-set fusion for distributed defects. The algorithm is able to adapt to a small number of defect image samples and high dimensionality of feature sets. Although machine learning-based object detection algorithms have overcome the shortcomings of traditional detection algorithms to some extent, such as poor robustness, they still have problems, such as large amounts of data, redundant information, and high feature space dimensions. They are also easily affected by multiple factors, such as environment, lighting, production processes, and noise.

In the research of steel surface defect detection based on deep learning methods. Liu et al. [16] proposed a new concurrent convolutional neural network with different image scales. The model uses only 20% of the steel surface data set as a training set and has higher detection accuracy. Nevertheless, the algorithm has the disadvantage of complex training. Zhao et al. [17] proposed an improved Faster R-CNN based on deformable convolution and multi-scale fusion training, which focuses on improving the detection ability of small targets, but the detection speed is slow. Cheng et al. [18] proposed an improved RetinaNet with a differential channel attention module and adaptive spatial feature fusion for steel surface defect detection. The model effectively fuses shallow and deep features to improve detection accuracy, but there are still shortcomings in feature extraction under complex background textures. Kou et al. [19] proposed an improved YOLOv3 model based on an anchor-free feature selection mechanism and dense convolution blocks. The model optimizes the detection performance at multiple scales by using an anchor-free mechanism, but has the disadvantage of high model complexity and high computational cost. Zhao et al. [20] proposed an improved YOLOv5 model based on dual feature pyramids and double decoupled heads, the model improves the detection accuracy of tiny defects on the surface of the steel, but there is a problem of model overfitting caused by the reuse of features. Liu et al. [21] proposed an improved YOLOv5 model based on a combined attention mechanism. This model introduces a cross-layer connection in the backbone network to reduce the problem of feature loss and introduces a cross-stage expansion weighted feature pyramid network in the neck network to improve the feature extraction ability of the model. However, this model does not consider the detection problem under complex texture interference. Yu et al. [22] proposed a two-stage network based on the squeeze excitation attention mechanism and dilated convolution. The network enhances the correlation of channel features, reduces noise interference to a certain extent, and expands the receptive field of the model, which is beneficial for improving the detection ability of small target defects. However, it is not designed for multi-scale targets. Fu et al. [23] proposed region-based fully convolutional networks, which improve the feature extraction ability of steel surface defects with unclear boundaries by introducing deformable convolution and attention mechanisms based on adaptive learning. However, due to the introduction of deformable convolutions and a two-stage network architecture, the detection speed of the network is slow, and the model volume is large. Wang et al. [24] proposed an improved YOLOX model, which introduces a coordinate attention mechanism in the backbone network to enhance feature extraction ability under complex textures and introduces varifocal loss to solve the problem of imbalance between positive and negative samples in the front. However, the varifocal loss requires the calculation of independent loss for each category, which increases the computational complexity and leads to a decrease in the model’s ability to detect defects in small samples. Zhang et al. [25] proposed a cross-scale weighted feature fusion network, which improves the bi-directional feature pyramid network by adding multiple cross-layer feature fusion paths and enhances the model’s ability to detect multi-scale objects. However, excessive feature fusion and scale matching increase much of the computational costs.

Based on the problems existing in the above algorithms, in order to improve the detection accuracy when dealing with the scale changes of surface defects on steel and the interference of background textures while meeting the deployment requirements of high speed and lightweight. This paper takes the one-stage object detection algorithm as the research direction and YOLOv5s algorithm as the baseline model. Additionally, it proposes an enhanced YOLOv5 model based on a larger kernel design and multi-path pyramid pooling structures. Our model achieved 76.8% mAP, 67.1 FPS, and 28.9 mb model volume on the NEU-DET dataset, which verified the effectiveness of the proposed model. The main contributions of this paper are as follows:

1. We propose a novel re-parameterized large kernel C3 (RepLK-C3) module with a large kernel design, which has a larger receptive field than the original C3 module and can pay more attention to shape features.
2. We revisit the effectiveness of using spatial pyramid pooling in deep layers of the network and remove the spatial pyramid pooling module in the last layer of the backbone network. Additionally, we re-focus on the importance of recognizing complex objects at every scale feature map, we redesigned the re-parameterized large kernel spatial pyramid pooling (RepLK-SPP) module with multiple large kernel designs. This module can extract strong positional features and use them for lateral propagation. By using this module after each scale feature map, we constructed the multi-path RepLK-SPP neck structure, which improves the model’s detection ability in complex textures.
3. We propose a training strategy that applies different kernel sizes to feature maps of different scales, allowing the network to capture the scale variations of steel surfaces to the greatest extent possible.

The rest of the paper is organized as follows: Section 2 describes the work related to the two major research directions studied in this paper. Section 3 provides a detailed description of the baseline model and the proposed network structure and related modules in this paper. Section 4 introduces the dataset and evaluation metrics used, and experimental results and analysis, validating the accuracy and effectiveness of the proposed algorithm. Section 5 gives the experimental conclusions and related analysis.

## 2. Related Work

### 2.1. Receptive Field

In the object detection model, expanding the receptive field of the model can help the model better capture the global information improving, so as to improve the model’s ability to detect tiny targets under complex texture interference. At present, in order to expand the receptive field of the model, some methods can be used, such as: 1. Increasing the number of layers of the model. By increasing the number of layers of the model, the receptive field of the model can be extended to a larger area; 2. Using global average pooling. Global average pooling can integrate the information of each position of the input image to produce a vector representing the entire image. 3. Using dilated convolution, dilated convolution can achieve a larger receptive field in the model by setting the expansion rate size; 4. Using large kernel convolution, which can enlarge the receptive field by equalizing the receptive field of the model with the size of the input image.

In the research of improving the receptive field for the detection of steel surface defects. Bi et al. [26] proposed an improved segmentation and decision network with dilated convolution. The network has improved detection accuracy in complex environments to a certain extent; however, its accuracy only improves by 1%. Tian et al. [27] proposed an improved CenterNet to increase the receptive field of the detector by extending the feature enhancement method and achieving the best trade-off between speed and accuracy; however, it may introduce additional noise interference while expanding feature information. Li et al. [28] proposed a receptive field enhanced YOLOv4 model for steel surface defect detection, which introduces a receptive field block to the path aggregation network for enhancing the information acquisition and feature extraction ability of the network. However, the receptive field block may not be able to obtain sufficient contextual information at the global level, which may affect the performance of the model in some complex scenarios. Zheng et al. [29] proposed a chained atrous spatial pyramid pooling network for steel surface defect detection. The network introduces multiple groups of atrous convolutions in series to achieve feature interaction and prominence; however, this leads to a large computational volume and may lead to overfitting problems.

In recent research, convolutional neural networks with large kernel designs have been revisited. Ding et al. [30] proposed a re-parameterized large kernel net (RepLKNet) with the idea of using re-parameterized convolutions to obtain larger receptive fields and more global information. However, when the kernel size gradually expands to 31 × 31 and above, there is a decrease in accuracy. Liu et al. [31] proposed a sparse large-kernel network with a 51 × 51 convolutional kernel. The network verified the ability to further improve the convolutional kernel size and eliminate the performance gap by strategically expanding the convolution; however, it does not consider the optimization problems and calculation speed brought by such a large core. Han et al. [32] proposed an improved UNet model. The model uses large kernels of depth-wise separable convolution to reduce parameters; however, the computational speed will significantly decrease when the kernel size of depth-wise separable convolution reaches a certain size. Yu et al. [33] proposed an InceptionNet, the network decomposes the large kernel depth-wise convolution along the channel dimension into four parallel branches, which improves the computational speed of large kernel CNNs almost without loss of performance; however, it only expanded the channel kernel to 16 and did not further discuss larger kernel designs. Although the above algorithm still has some problems, the performance of larger kernel design in upstream tasks still has significant advantages compared with other methods. Therefore, our study aims to improve the model’s receptive field by enlarging the kernel of convolution and optimizing some existing problems so as to explore high-precision detection of surface defects in steel.

### 2.2. Multi-Scale Defect Detection

Multi-scale feature fusion is one of the key research fields of computer vision, which can significantly improve the accuracy of model detection for different scale targets. Lin et al. [34] proposed a feature pyramid network (FPN) model to solve the problem of difficulty in multi-scale detection; however, this method only transfers the multi-scale features of the backbone network and does not consider the deeper feature fusion design. Zhao et al. [35] proposed a pyramid pooling module to solve the difficulty of understanding complex scenes, which integrates features of different scales to explore global contextual information. However, it only introduces pyramid pooling modules in the deepest layer of the backbone network without considering multi-scale receptive field design for other scales. Chen et al. [36] proposed an atrous spatial pyramid pooling module. The model uses multiple sampling rates to capture feature information at multiple scales in order to capture contextual information of the target object. However, using atrous convolution will lead to the problem of feature information overlap when dealing with large targets, which affects the accuracy of the model. Zhang et al. [37] proposed the backbone network architecture of efficient pyramid squeeze attention, with stronger multi-scale representation ability. However, it only performs multi-scale feature extraction on channels and does not consider spatial feature information. Tang et al. [38] proposed a layered multiscale network suitable for cross-scale visual defect detection and constructed a new backbone network called a hierarchical multi-scale network; however, the implementation is achieved by stacking through grouped convolution, when the number of groups is large, spatial information loss may occur.

In the research of multi-scale feature fusion for steel surface defect detection. Yeung et al. [39] proposed a detection model of fusion attention framework, which is used to enhance the discrimination ability of different scale defect detection through adaptive balance feature fusion and has a good performance on both NEU-DET and GC10-DET data sets. However, the feature extraction for multi-scale defects relies on the stacking of multiple deformable convolutions, which brings a large amount of computation. Liu et al. [40] proposed a multi-scale context defect network, and it improves the detection ability of multi-scale defects by introducing multiple parallel expansion convolutions and uses multiple parallel feature enhancement and selection modules to improve feature extraction ability. However, this brings a large amount of computation, and its detection speed in the NEU-DET dataset is only 14.1 FPS. Guo et al. [41] proposed an improved YOLOv5 network, which adds a transformer block to the backbone network and prediction head, combining features with global information to enhance the detector’s dynamic performance for objects of different scales. However, the transformer blocks have a large computational cost, which reduces the inference speed of the model.

The above research on multi-scale defect detection of steel surface almost all focus on improving the extraction ability of backbone network for multi-scale features; however, they do not pay attention to the design direction of different receptive fields. In this paper, we use a multi-path spatial pyramidal pooling feature fusion path to fully extract the feature information of each scale feature map and participate in lateral propagation, which significantly improves the detection capability of the model for multi-scale defects.

## 3. Methods

### 3.1. Review of YOLOv5

**Figure 1:**
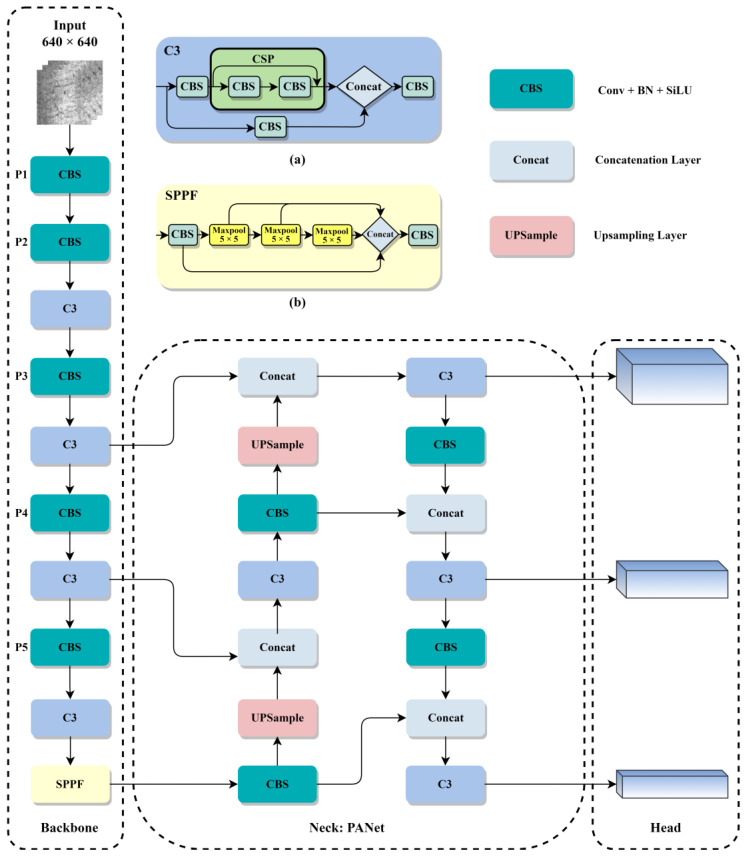
Structure of YOLOv5: (**a**) C3 module; (**b**) Spatial pyramid pooling fast module.

YOLOv5 [42] is a one-stage target detection network with the advantages of high accuracy, high speed and light weight. It can be divided into four versions based on the depth and width of the network: YOLOv5s, YOLOv5m, YOLOv5l, and YOLOv5x. Among them, YOLOv5s has the smallest model size and fastest detection speed and is widely used in industrial detection fields. It consists of four parts: Input, Backbone, Neck, and Head network. The structure of YOLOv5 is shown in Figure 1.

The Input network includes three parts: mosaic data augmentation, adaptive anchor box calculation, and adaptive image scaling. Mosaic data augmentation combines four images using random cropping, scaling, and shuffling methods, which is beneficial for detecting small objects. For different datasets, the adaptive anchor box calculation method generates anchor frames of different scales. The adaptive image scaling method can resize the input image to the specified size.

The backbone network contains multiple CBS modules, which reduce the size to ½ of the original size each time and contain a total of five size feature maps of P0-P5. The C3 module, as shown in Figure 1a, consists of three conv- and cross-stage partial (CSP) structures and residual connections, which can enhance feature extraction ability while ensuring the model is lightweight. The spatial pyramid pooling fast (SPPF) module is shown in Figure 1b. It consists of a CBS module and several maxpooling layers, which fuse the feature maps of different receptive fields and enrich the expression ability of feature maps.

The neck network combines the Feature Pyramid Network (FPN) with the Path Aggregation Network (PANet) to fuse the semantic information extracted by deep networks with the positional information extracted by shallow networks. At the same time, the feature fusion between the backbone and neck makes the model acquire richer feature information and sends it to the head to output the prediction results.

The head network and anchor boxes of different sizes are used to predict and classify feature maps of different scales. In the prediction stage, multiple prediction boxes with confidence are generated for a single target. According to the confidence level, the non-maximum suppression (NMS) algorithm selects the optimal predicted box and leaves the optimal target box.

### 3.2. Improved Network Architecture

**Figure 2:**
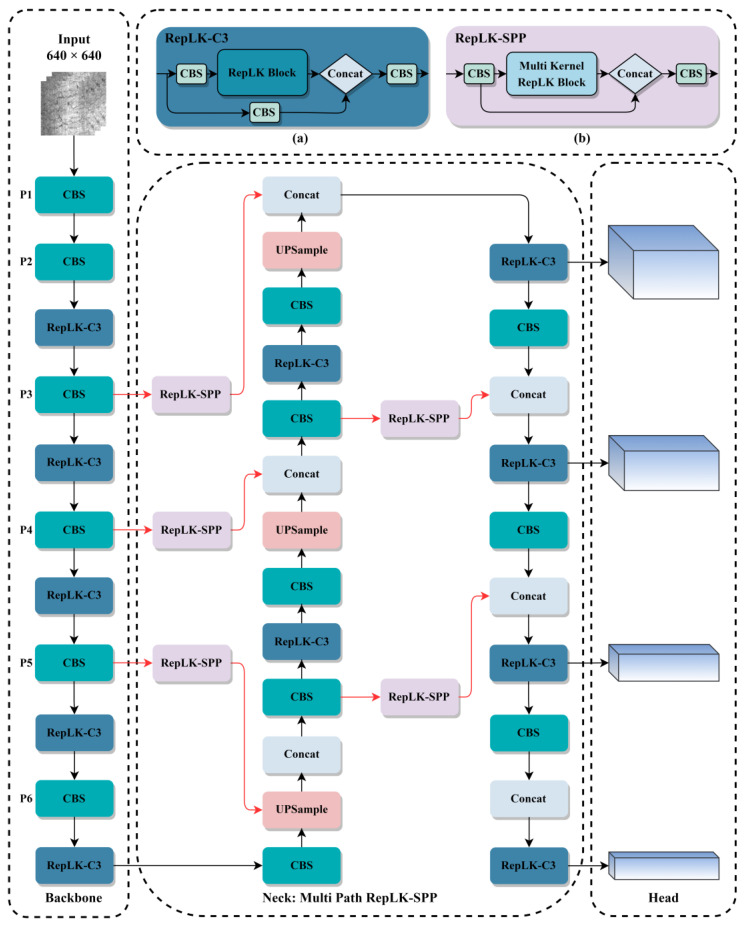
Structure of improved network architecture: (**a**) Re-parameterized large kernel C3 module; (**b**) Re-parameterized large kernel spatial pyramid pooling module.

To solve the problem of missed and false detection caused by a large number of tiny targets and complex background textures on the steel surface, based on the original YOLOv5s, the network structure has been redesigned and improved, as shown in Figure 2. The improvements are as follows.

1. According to statistics, there are a large number of large-scale, tiny textured defect images in the NEU-DET steel surface dataset. Therefore, we added an additional P6 layer with a smaller size to the backbone network and integrated it into the neck network. We also added a detection head to the head network to better adapt to multi-scale defect object detection.
2. Due to the interference of texture features on the surface of the steel, there are a large number of defects with subtle features. Therefore, in order to increase the receptive field of the model, we combine the re-parameterized large kernel convolution block with the C3 module, replace the original CSP layer, and introduce the newly designed re-parameterized large kernel C3 (RepLK-C3) module into the backbone and neck networks. The structure of RepLK-C3 is shown in Figure 2a.
3. In order to improve the detection accuracy of multi-scale defects on the surface of the steel, we design a re-parameterized large kernel spatial pyramid pooling (RepLK-SPP) module with different receptive fields, which replaces maxpooling layers with re-parameterized large kernel convolutions and changes the connection structure to parallel architecture. The structure of RepLK-C3 is shown in Figure 2b.
4. We revisit the effects of using SPPF modules in the deeper layers of the network and refocus on multi receptive field feature extraction for each scale feature map. Based on this idea, we propose a multi-path re-parameterized large kernel spatial pyramid pooling feature fusion path in the neck network. Specifically, we removed the SPPF module from the bottom layer of the backbone network and used the newly proposed RepLK-SPP module to connect the down-sampled feature maps after the CBS module. This connection is marked with a red line in Figure 2.

### 3.3. Re-Parameterized Large Kernel Convolution C3 Module

**Figure 3:**
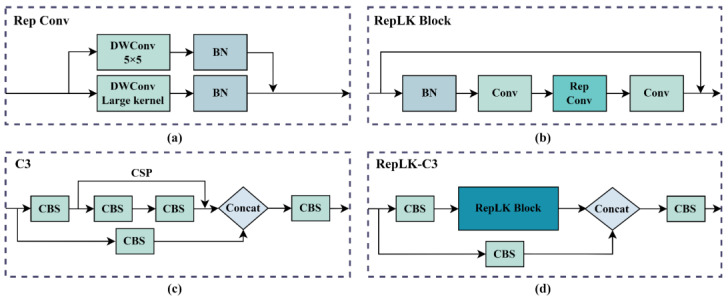
Illustration of the RepLK-C3 module: (**a**) Rep Conv module; (**b**) RepLK Block; (**c**) C3 module; (**d**) RepLK-C3 module.

In the backbone network of YOLOv5s, by continuously using the CBS module and C3module for down-sampling and feature extraction, feature maps of different scales are obtained for feature fusion. By visualizing these feature maps, we observed that, while extracting effective features, a large number of ineffective texture features are not effectively filtered. RepLKNet [30] proposed a large kernel structure called Replk Block, which demonstrated that using large kernel re-parameterized convolutions can lead to larger effective receptive fields and higher shape bias. The re-parameterized large kernel conv is shown in Figure 3a. It contains a small kernel and a large kernel depth separable convolution and batch normalization. By connecting the large kernel and small kernel in a re-parameterized way, the memory computation cost can be reduced while enlarging the kernel size. RepLK module is shown in Figure 3b, which consists of batch normalization, two 1 × 1 convolutions, re-parameterized convolution, and residual connection. To solve the problem of interference of texture features, we combine the RepLK module with the C3 module in YOLOv5s, replacing the CSP structure to obtain a new RepLK-C3 module. The structure of this module is shown in Figure 3d. The addition of the large kernel re-parameterized conv allows the RepLK-C3 module to obtain a larger receptive field and pay more attention to the shape features within the region when extracting features.

**Figure 4:**
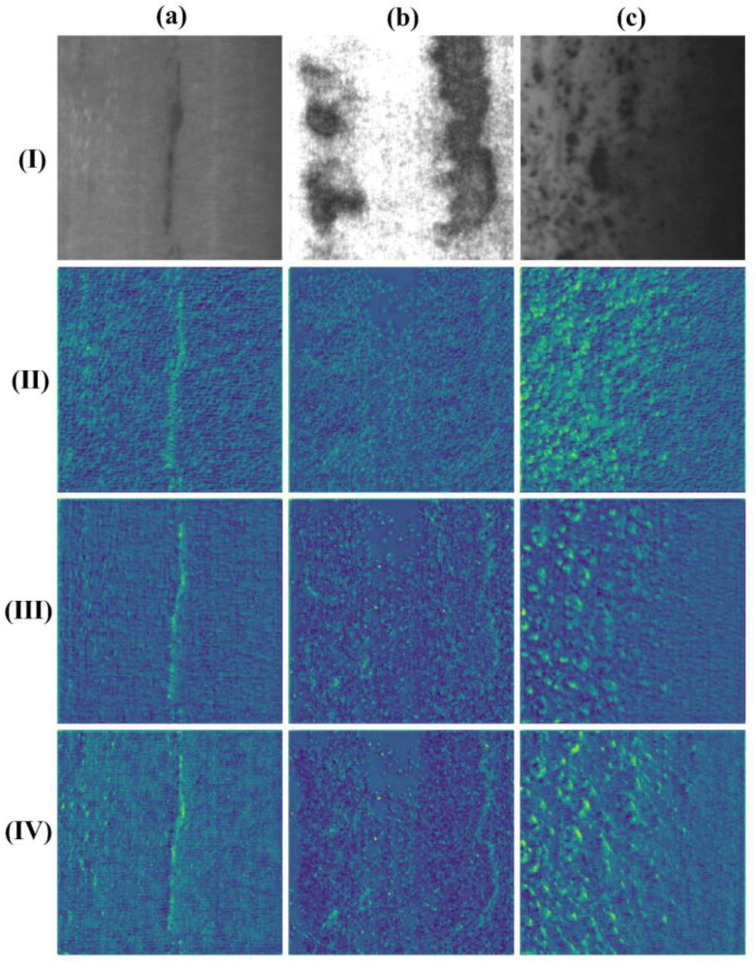
RepLK-C3 module feature map visualization: (**I**) Original image; (**II**) Results of C3 module; (**III**) Results of RepLK-C3 module with 15 × 15 kernel size; (**IV**) Results of RepLK-C3 module with 41 × 41 kernel size; (**a**) Inclusions; (**b**) Patches, and (**c**) Rolled-in scale.

In order to show the effectiveness of the RepLK-C3 module for extracting shape features, we replaced the C3 module at the P2 layer with RepLK-C3 module by adding it to the backbone network structure and visualize the feature maps extracted by the RepLK-C3Module with a small-size convolution kernel (15 × 15) and a large-size convolution kernel (41 × 41), respectively, and compare them with the feature maps output by the C3module in the original position. Selected three types of defects with significant shape features in the steel surface dataset, including inclusion, patches, and pitted surface, for feature map visualization, as shown in Figure 4. The results show that the RepLK-C3 module with large convolution kernels can bring larger receptive fields while effectively eliminating texture features and can better focus on shape features. Moreover, the ability to extract shape features will be continuously strengthened with the deepening of network layers.

### 3.4. Multi-Path RepLK-SPP Neck

**Figure 5:**
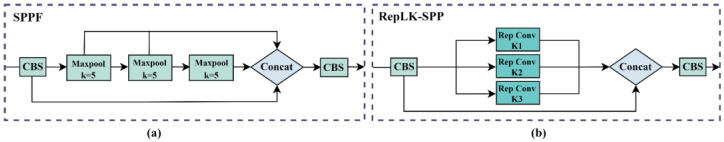
Illustration of the RepLK-SPP module: (**a**) SPPF module; (**b**) RepLK-SPP module.

In the backbone network of YOLOv5s, the spatial pyramid pooling fast module is located in the last layer. Its combination of multiple small-sized pooling layers replaces the single large-sized pooling layers in the SPP module. Therefore, while retaining fusing feature maps of different receptive fields and enriching the expressive ability of feature maps, the running speed is further improved. The structure of the SPPF module is shown in Figure 5a. The module first uses a 1 × 1 convolution operation, then uses three maxpooling layers with a window size of 5 to extract features with different receptive fields. Then, these four processed features will be fused and processed for channel number through a 1 × 1 convolution. At the beginning of the design of the SPPF module, it was believed that aggregating information from the previous layers that had already been deeply processed may be more in line with the human brain’s cognitive process of objects when processing objects of different scales at deeper layers. We revisit this view when the feature information of the object passes through multiple feature extraction layers, its feature map has been reduced to 1/32 of the original, the feature information contained is relatively little, and the feature information already belongs to relatively high-level abstract information, on this basis, using the SPPF module to fuse the feature information of multiple receptive fields may not bring the expected effect. Therefore, we propose a multi-path spatial pyramid feature fusion structure, as shown in Figure 5. Using spatial pyramid pooling module to extract multi-scale features from each down-sampled feature map and pass them to deeper layers of the network. Our experimental results show that for large feature maps, a larger receptive field should be used when using spatial pyramid pooling after different scale feature maps. Based on this idea, we replace the max pooling layers in the SPPF module with the Rep Conv module with a larger receptive field, which can avoid the disadvantage of information loss caused by the pooling operation itself. In addition, Rep Conv can implement larger kernels without incurring excessive memory computation costs. Therefore, it is possible to directly design and construct the RepLK-SPP module according to the parallel architecture of the SPP module without the need for grouping stacking with a small field of view, such as the SPPF module, as shown in Figure 5b.

**Figure 6:**
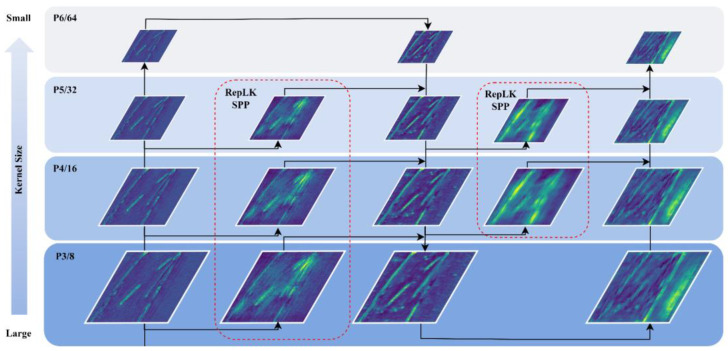
Structure of multi-path RepLK-SPP neck, the feature map enclosed by the red dashed box is generated by the RepLK-SPP module, while the others are generated by the RepLK-C3 module. The feature maps at the same scale layer are designed with the same kernel size, and the kernel size will decrease as the feature map size decreases.

**Figure 7:**
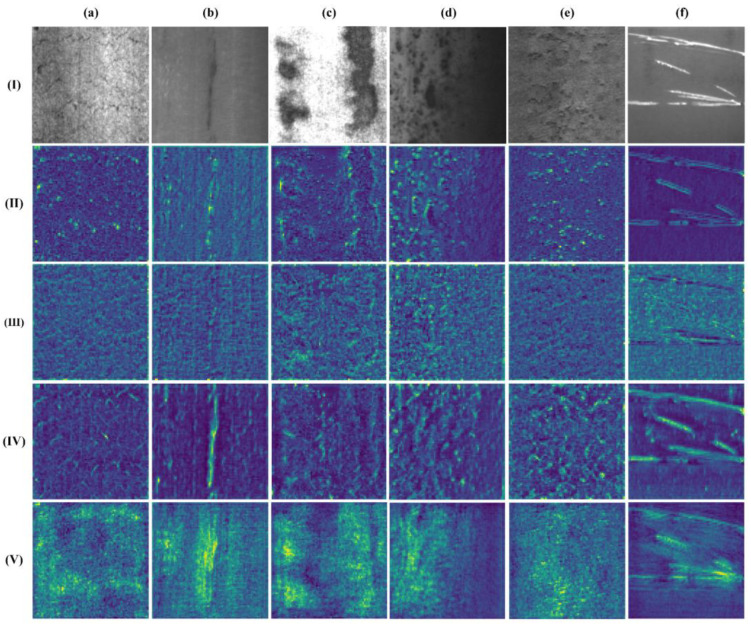
Feature map visualization: (**I**) Original image; (**II**) Results of CBS module of P3 Layer; (**III**) Results of C3 module; (**IV**) Results of RepLK-C3 module; (**V**) Results of RepLK-SPP module; (**a**) Crazing; (**b**) Inclusion; (**c**) Patches; (**d**) Pitted surface; (**e**) Rolled-in scale; and (**f**) Scratches.

As shown in Figure 6, in the multi-path spatial pyramid feature fusion structure designed in this paper, the same down-sampled feature map will be processed separately by the RepLK-C3 module and the RepLK-SPP module. Additionally, we use the same kernel size design for feature maps of the same size, with the kernel size changing as the feature map scale changes, in order to achieve the best perception field. We visualized the feature maps of the CBS module of the third down-sampling, the Rep-C3 module, the Rep-SPP module in the subsequent branch, and the corresponding position of the C3 module in the original YOLOv5. The results are shown in Figure 7. It can be seen from (III and IV) that compared with the C3 module, the RepLK-C3 module proposed in this paper can filter irrelevant texture information and highlight shape information while retaining more detailed information; From (V), it can be seen that the proposed RepLK-SPP module can greatly highlight the position information of the image, especially for defect types, such as crazing, pitted surface, and rolled-in scale that contain a large amount of complex background interference. In the original feature fusion path design, the feature map after the feature extraction of the C3 module will be sent to the subsequent network. However, for the processing of complex textures on the steel surface, the C3 module is not very competent, which will bring poor results to the subsequent feature fusion. Therefore, by using a branch design to extract shape and position information separately using the RepLK-C3 module and RepLK-SPP module on each scale feature map, significant effects can be achieved.

## 4. Experiment

### 4.1. Dataset

**Figure 8:**
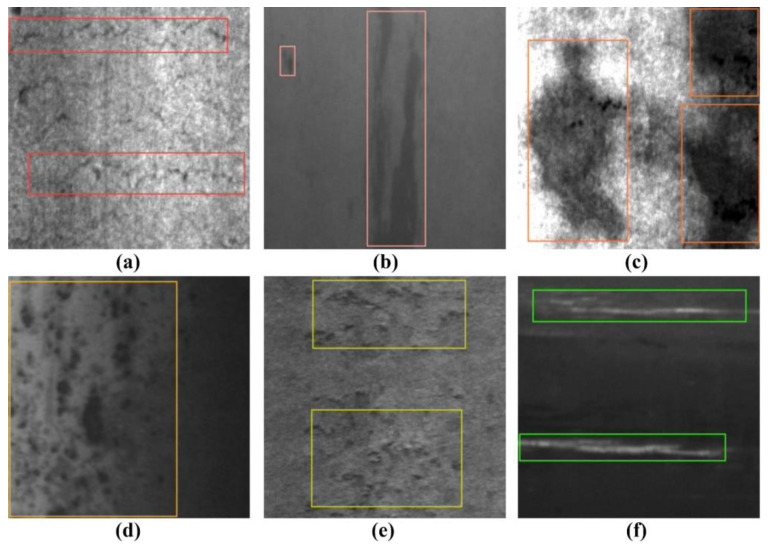
Six types of surface defects in the NEU-DET dataset: (**a**) Crazing; (**b**) Inclusion; (**c**) Patches; (**d**) Pitted surface; (**e**) Rolled-in scale; and (**f**) Scratches.

The experiment in this paper uses the hot-rolled strip surface defect dataset (NEU-DET) [43] collected by the School of Mechanical Engineering and Automation of Northeastern University, which contains six types of surface defects of the hot-rolled strip, as shown in Figure 8, including crazing (Cr), inclusion (In), patches (Pa), pitted surface (Ps), rolled-in scale (RS), and scratches (Sc). The dataset contains 1800 grayscale images of steel surface defects, with 300 images for each type of defect and a resolution of 200 × 200 for each image. The training set, validation set, and test set are divided in the ratio of 8:1:1, as shown in Table 1, with 1440 images in the training set and 180 images in both the test set and validation set.

### 4.2. Training Environment and Parameters

This experiment is built on an AutoDL server with an RTX3090 GPU, Intel(R)Xeon(R)Platinum 8358P CPU, running on a Linux operating system, using PyTorch1.8.1, Python 3.8, and CUDA 11.0. The experimental environment was set up as shown in Table 2.

To train our models, the Stochastic Gradient Descent (SGD) optimizer was used with a momentum and weight decay of 0.937 and 0.0005, respectively. Additionally, the learning rate was adjusted to 0.01. To find the optimal hyperparameter values, we chose image size 640 × 640, batch size 32, and the model was run for up to 300 epochs. The training parameters are shown in Table 3.

**Figure 9:**
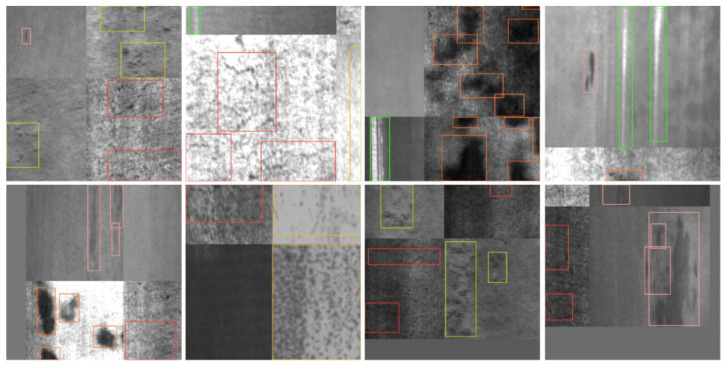
Example images of mosaic enhancement.

In order to prevent overfitting and improve the generalization of the model, we use image enhancement methods, such as HSV enhancement, random scaling sampling, random flipping, and mosaic enhancement, during the training process. These methods can greatly enhance the diversity of data and effectively improve the model’s detection ability for small targets. The hyperparameters of image enhancement are shown in Table 4. The mosaic data enhancement is shown in Figure 9.

### 4.3. Evaluation Metrics

To evaluate the overall performance of the model, the evaluation metrics used in this experiment are precision (P), recall (R), average precision (AP), mean average precision (mAP), FPS, giga floating-point operations per second (GFLOPS), parameters, and model volume. Their calculations are shown in Equations (1)–(4).

AP represents the area enclosed by the P-R curve, where P, R, and the calculation expressions are shown below.

(1) $$Precision=\frac{TP}{TP+FP}$$

(2) $$Recall=\frac{TP}{TP+FN}$$

In the above equation, True Positive (TP) represents the samples that are judged as positive and correct, False Positive (FP) represents the negative samples that are judged as positive, and False Negative (FN) represents the positive samples that are judged as negative.

The calculation formula for AP and Map are as follows:

(3) $$AP=\int_{0}^{1}P(R)dR$$

(4) $$mAP=\frac{\sum_{j=1}^{S}\;AP(j)}{S}$$

In the above equation, S represents the number of all categories and is both the denominator and the sum of the AP of all categories.

Additionally, FPS represents the number of images the model can detect per second; the higher FPS, the faster the detection speed of the model. GFLOPS represents the number of floating-point operations that a model can perform per second; the higher the GFLOPs, the higher the computational complexity of the model. Parameters usually refer to the number of trainable parameters in a model, including the number of layers in a network and the weights of each layer. Model volume refers to the size of its model weight file, which is determined by the number and data type of all trainable parameters in the model. The unit of model volume is mb.

### 4.4. Experiment of Multi Kernel Size Re-Parameterized Large Kernel C3 Module

In order to evaluate the influence of RepLK-C3 modules with different kernel sizes on the model detection accuracy, we designed relevant experiments by introducing five sets of RepLK-C3 modules with convolution kernel sizes of 9 × 9, 15 × 15, 25 × 25, 35 × 35, and 41 × 41, and two groups of mixed-size convolution kernel RepLK-C3 modules. A control group without RepLK-C3 modules was also introduced, which adopted an architecture fused with the P6 layer. The experimental schemes and results are shown in Table 5 and Table 6, respectively.

The experimental results show that as the size of the convolution kernel increases, there is not a significant change in the volume of the model, only a small increase in computational parameters. This is because depth-wise separable convolution has the characteristic of a small computational cost. Moreover, the detection accuracy of the model generally increases with the enlargement of the size of the convolution kernel, and the model achieves the best mAP when the convolution kernel size is 25 × 25. However, when the size of the kernel is beyond 25 × 25, the mAP decreases, and the oversized convolution kernel size may not be suitable for smaller feature maps in the P6 layer. Therefore, we propose a backbone network with mixed-size kernels to address this issue, and the size of the kernel decreases gradually in order to adapt to variances in the feature map scale. Two sets of experiments were designed, as shown in Schemes 6 and 7, with the kernel size decreasing from 25 × 25 to 5 × 5 and from 41 × 41 to 9 × 9, respectively. The experimental results of Scheme 7 show that using a mixed-size convolution kernel with a larger kernel size in the RepLK-C3 module can further improve detection accuracy and effectively reduce the number of parameters.

### 4.5. Experiment of Multi-Path RepLK-SPP Neck

To verify the effectiveness of the proposed multi-path RepLK-SPP feature fusion neck and to explore the impact of the size of the k1, k2, and k3 kernels in the RepLK-SPP module on the accuracy of model detection, we designed relevant experiments as shown in Table 7. Scheme 1 is a control experiment that introduces multi-path RepLK-SPP feature fusion neck but does not contain a RepLK-SPP module. Schemes 2–4 are experiments that use different sizes of k1, k2, and k3 of the RepLK-SPP module accordingly. The experiment is based on the network that introduces the P6 layer and the mixed kernel RepLK-C3 module. Moreover, we still adopt the same kernel size design strategy for the feature map of the same layer. The experimental results are shown in Table 8.

According to the results of Scheme 1 in Table 8, after the introduction of multi-path RepLK-SPP feature fusion neck, even if the receptive field is not designed for the feature map, a certain accuracy improvement is still obtained; According to the results of Scheme 2 in Table 6, after the introduction of the RepLK-SPP module with a smaller kernel, the model did not achieve better results, which indicates that a smaller receptive field cannot effectively capture positional information; According to the results of Schemes 3–5 in Table 6, increasing the kernel size of RepLK-SPP can significantly improve the detection accuracy of the model, and the mixed kernel design with the large receptive field can extract the location information features under complex texture interference to the maximum extent. When the kernel design is (25, 35, 41), (15, 25, 35), and (9, 15, 25), the model reaches the best mAP: 76.8% and has the best precision: 76.1%. In addition, through the comparison of Parameters and giga floating-point operations per second (GFLOPS), the proposed multi-path RepLK-SPP feature fusion neck in this paper does not significantly increase the number of parameters and model complexity.

### 4.6. Comparison of Different Algorithms

**Figure 10:**
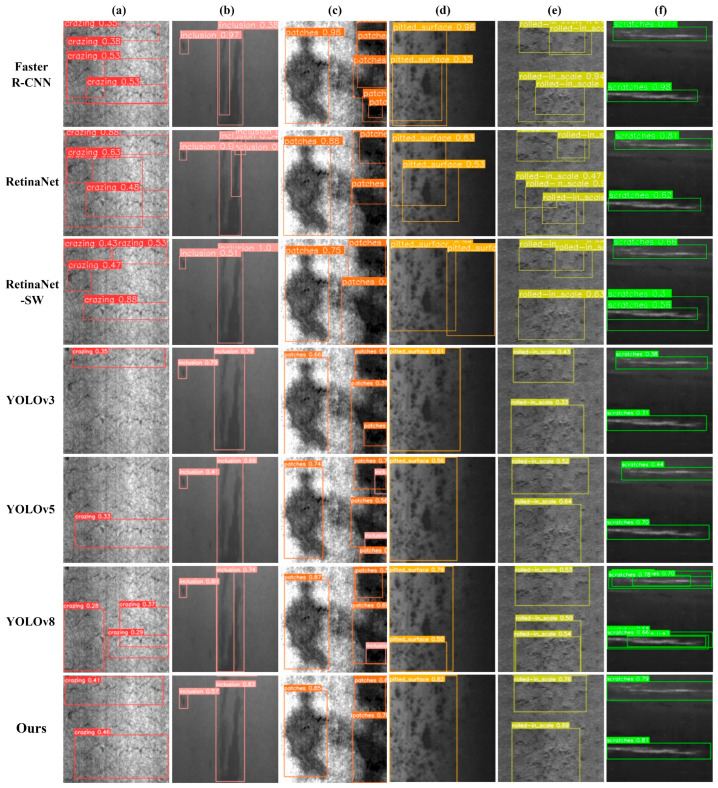
Detection results of different algorithms: (**a**) Crazing; (**b**) Inclusion; (**c**) Patches; (**d**) Pitted surface; (**e**) Rolled-in scale; and (**f**) Scratches.

To verify the detection precision and reliability of the proposed model in this paper, Faster R-CNN [17], RetinaNet [44], RetinaNet-SW (RetinaNet with Swin transformer backbone) [45], YOLOv3 [46], YOLOv5 [42], and the latest YOLOv8 [47] network models were used for comparison. All models were tasked with detection on the NEU-DET steel surface dataset, and the detection results are shown in Figure 10. The comparison experimental data are shown in Table 9. According to the detection results, the proposed model effectively improves the detection precision for various types of defects compared to several mainstream models. Compared to the original YOLOv5, The AP values of our model for the detection of three types of defects with prominent shape features: inclusion, patches, and scratches are 87.5%, 92.4%, and 77.5%, respectively, which indicates that the RepLK-C3 module can pay more attention to the shape features. As for other mainstream algorithms, this model improves the AP values of rolled-in scale and pitted surface with complex texture feature interference and weak feature, reaching 82.0% and 68.2%, respectively, which indicates that the multi-path RepLK-SPP module can extract effective location information in complex scenarios. In terms of model performance, the mAP value of our model reaches 76.8%, compared to the original YOLOv5 and the latest YOLOv8 algorithm; these are increases of 8.6% and 3.7%, respectively. At the same time, the model volume is 28.9 mb, compared with RetinaNet-SW 282.0 mb, RetinaNet 277.0 mb, YOLOv3 117.0 mb, and Faster R-CNN 41.2 mb, it can still meet the lightweight deployment requirements; Meanwhile, our model has a high detection speed, reaching 67.1 FPS, which is higher than RetinaNet 63.4 FPS, RetineNet-SW 46.1 FPS and Faster R-CNN 23.4 FPS, and realizes real-time detection.

According to the detection results in Figure 10, Retinanet has a large number of false detections in instance samples, while Retinanet-SW, which introduces the Swin Transformer backbone network, can better extract global features but still has a certain degree of false detections. Faster R-CNN has high detection accuracy, but there are a large number of overlapping boxes during detection, which is not conducive to actual industrial detection needs. YOLOv3 has a certain degree of missed detection and lower confidence. In addition, YOLOv5 and YOLOv8 have false detection problems in the detection of Patches class samples. Only the model proposed in this paper can achieve accurate detection without missed detection, false detection, and interference from complex textures. Therefore, it can be concluded that our model is superior to other mainstream target detection algorithms in terms of model volume, detection accuracy, and detection speed and can be better applicable to the defect detection task under complex texture interference on the surface of steel.

## 5. Conclusions

This paper proposes a model which improves on the YOLOv5 network structure based on the characteristics of the NEU-DET steel surface dataset. (1) Introducing the re-parameterized large kernel C3 (RepLK-C3) module, which pays more attention to shape features, replaced the C3 module to improve the model’s detection performance of significant defects in shape features. Moreover, the expansion of the kernel can enhance this capability to a certain extent. (2) Proposing a re-parameterized large kernel spatial pyramid pooling (RepLK-SPP) module with a large receptive field and introducing them in each down-sampling feature map. Then, the multi-path RepLK-SPP feature fusion neck with different kernel sizes is designed for the scale of the feature map. Experimental results show that the module and the proposed feature fusion path can extract more significant location information in complex backgrounds. (3) Proposing a training strategy that uses different kernel sizes based on the scale changes of feature maps. Experimental results show that this strategy can make the model’s receptive field better fit the scale changes of the target object and can reduce the parameter consumption caused by large kernels.

The experiment on the NEU-DET dataset shows that the mean average precision value reaches 76.8%, compared with the YOLOv5s and YOLOv8s, which increased by 8.6% and 3.7%, respectively. Meanwhile, the model volume increased slightly to 28.9 mb, which is still able to meet the demand for lightweight deployment compared to RetinaNet 277.0 mb, RetinaNet-SW (RetinaNet with Swin transformer Backbone) 282.0 mb, YOLOv3 117 mb, and Faster R-CNN 41.2 mb. At the same time, the detection speed is 67.1 FPS, which meets the industrial demand for steel surface defect detection. Additionally, our model improved the detection accuracy of crazing and rolled in-scale, which contain a large number of weak texture features and are densely distributed by 14.4% and 11.1%, respectively. Additionally, the detection accuracy of inclusion and scratch defects with prominent scale changes and significant shape features was improved by 10.5% and 6.6%, respectively. Currently, we have designed a mixed receptive field for multi-scale defects of steel surfaces in our experiments. In future work, we will further explore the application of the mixed receptive fields model in other related fields.

## Figures and Tables

**Table 1:** Dataset division.

Data	Samples
Training	1440
Validation	180
Test	180

**Table 2:** Experimental environment.

Experimental Environment	
Processor	Intel(R)Xeon(R)Platinum8358P CPU
Operating system	Linux
Ram	32GB
Graphics card	RTX3090 GPU
Programming language	Python3.8
Deep learning libraries	PyTorch1.8.1
Deep learning toolkit	CUDA11

**Table 3:** Training parameters.

Parameter	Value	Parameter	Value
Learning Rate	0.01	Weight Decay	0.0005
Batch Size	32	Momentum	0.937
Image Size	640 × 640	Epoch	300
Dataloader	16	Optimizer	SGD

**Table 4:** Augmentation hyperparameters.

Parameter	Value
HSV-Hue	0.015
HSV-Saturation	0.7
HSV-Value	0.4
Image scale	0.5
Image flip left-right	0.5
Mosaic	1.0

**Table 5:** Experimental schemes of multi-kernel size RepLK-C3 module.

Schemes	P2	P3	P4	P5	P6
1	3 × 3	3 × 3	3 × 3	3 × 3	3 × 3
2	9 × 9	9 × 9	9 × 9	9 × 9	9 × 9
3	15 × 15	15 × 15	15 × 15	15 × 15	15 × 15
4	25 × 25	25 × 25	25 × 25	25 × 25	25 × 25
5	35 × 35	35 × 35	35 × 35	35 × 35	35 × 35
6	41 × 41	41 × 41	41 × 41	41 × 41	41 × 41
7	33 × 33	23 × 23	13 × 13	9 × 9	7 × 7
8	35 × 35	25 × 25	15 × 15	9 × 9	7 × 7
9	41 × 41	35 × 35	25 × 25	15 × 15	9 × 9

**Table 6:** Experimental results of multi-kernel size RepLK-C3 module.

Schemes	Precision (%)	Recalls (%)	mAP@0.5 (%)	Parameters	GFLOPS (G)
1	69.6	66.4	70.7	12,337,892	16.3
2	65.0	70.0	69.9	12,976,356	16.7
3	63.9	71.7	72.4	13,294,308	17.6
4	68.3	72.2	72.3	14,177,508	20.3
5	63.6	72.6	71.0	15,502,308	24.2
6	65.8	68.6	70.5	16,509,156	27.2
7	66.2	71.4	70.6	13,115,108	19.4
8	73.4	65.5	72.0	13,170,660	20.2
9	62.7	74.3	72.8	13,645,540	23.1

**Table 7:** Experimental schemes of different size convolution kernel RepLK-SPP module.

Schemes	P3	P4	P5
1	5, 5, 5	5, 5, 5	5, 5, 5
2	9, 15, 25	7, 9, 15	5, 7, 9
3	15, 25, 35	9, 15, 25	7, 9, 15
4	9, 35, 41	9, 25, 35	9, 15, 25
5	25, 35, 41	15, 25, 35	9, 15, 25

**Table 8:** Experimental results of multi-kernel size RepLK-SPP module.

Schemes	Precision (%)	Recall (%)	mAP@0.5 (%)	Parameters	GFLOPS (G)
1	71.4	69.2	73.2	14,027,684	24.9
2	71.1	66.7	73.3	14,256,868	26.1
3	75.0	69.5	75.1	14,504,676	27.4
4	69.9	73.3	75.6	14,739,364	28.9
5	76.1	70.7	76.8	14,905,572	29.7

**Table 9:** Comparison of different algorithms.

Types		Faster R-CNN	RetinaNet	RetinaNet-SW	YOLOv3	YOLOv5	YOLOv8	OURS
AP (%)	CR	54.5	50.8	50.2	40.7	38.5	53.9	52.9
	IN	81.1	77.8	77.3	78.0	77.0	78.0	87.5
	PA	89.3	89.5	87.7	90.5	90.7	90.4	92.4
	PS	79.8	80.4	84.5	78.7	75.0	89.0	82.0
	RS	62.5	62.8	62.4	52.3	57.1	42.1	68.2
	SC	82.6	55.3	72.5	71.4	70.9	85.2	77.5
mAP@0.5 (%)		75.0	69.5	72.4	68.6	68.2	73.1	76.8
FPS (f/s)		23.4	63.4	46.1	75.2	133.3	117.6	67.1
Parms (Mb)		41.2	277.0	282.0	117.0	13.8	21.5	28.9

## Data Availability

The NEU-DET is available online at http://faculty.neu.edu.cn/songkechen/zh_CN/zdylm/263270/list/index.htm (accessed on 15 April 2023).

## References

[1] Mordia R., Verma A.K. (2022). Visual techniques for defects detection in steel products: A comparative study. Eng. Fail. Anal..

[2] Tang B., Chen L., Sun W., Lin Z. (2023). Review of surface defect detection of steel products based on machine vision. IET Image Process..

[3] Ramírez I.S., Márquez F.P.G., Papaelias M. (2023). Review on additive manufacturing and non-destructive testing. J. Manuf. Syst..

[4] Czimmermann T., Ciuti G., Milazzo M., Chiurazzi M., Roccella S., Oddo C.M., Dario P. (2020). Visual-Based Defect Detection and Classification Approaches for Industrial Applications—A Survey. Sensors.

[5] Xu M., Yoon S., Fuentes A., Park D.S. (2023). A Comprehensive Survey of Image Augmentation Techniques for Deep Learning. Pattern Recognit..

[6] Guo Z., Shuai H., Liu G., Zhu Y., Wang W. (2022). Multi-level feature fusion pyramid network for object detection. Vis. Comput..

[7] Guo M., Xu T., Liu J., Liu Z., Jiang P., Mu T., Zhang S., Martin R.R., Cheng M., Hu S. (2022). Attention mechanisms in computer vision: A survey. Comput. Vis. Media.

[8] Vaswani A., Shazeer N., Parmar N., Uszkoreit J., Jones L., Gomez A.N., Kaiser Ł., Polosukhin I. (2017). Attention is all you need. Adv. Neural Inf. Process. Syst..

[9] Usamentiaga R., Lema D.G., Pedrayes O.D., Garcia D.F. (2022). Automated surface defect detection in metals: A comparative review of object detection and semantic segmentation using deep learning. IEEE Trans. Ind. Appl..

[10] Wen X., Song K., Huang L., Niu M., Yan Y. (2019). Complex surface ROI detection for steel plate fusing the gray I mage and 3D depth information. Optik.

[11] Xu K., Liu S., Ai Y. (2015). Application of Shearlet transform to classification of surface defects for metals. Image Vis. Comput..

[12] Yan X., Wen L., Gao L. (2019). A fast and effective image preprocessing method for hot round steel surface. Math. Probl. Eng..

[13] Chu M., Liu X., Gong R., Liu L. (2018). Multi-class classification method using twin support vector machines with multi-information for steel surface defects. Chemom. Intell. Lab. Syst..

[14] Yue X., Ma G., Liu F., Gao X. (2021). Research on image classification method of strip steel surface defects based on improved Bat algorithm optimized BP neural network. J. Intell. Fuzzy Syst..

[15] Wang Y., Xia H., Yuan X., Li L., Sun B. (2018). Distributed defect recognition on steel surfaces using an Improved random forest algorithm with optimal multi-feature-set fusion. Multimed. Tools Appl..

[16] Liu Y., Yuan Y., Balta C., Liu J. (2020). A light-weight deep-learning model with multi-scale features for steel surface defect classification. Materials.

[17] Zhao W., Chen F., Huang H., Li D., Cheng W. (2021). A new steel defect detection algorithm based on deep learning. Comput. Intell. Neurosci..

[18] Cheng X., Yu J. (2020). RetinaNet with difference channel attention and adaptively spatial feature fusion for steel surface defect detection. IEEE Trans. Instrum. Meas..

[19] Kou X., Liu S., Cheng K., Qian Y. (2021). Development of a YOLO-V3-based model for detecting defects on steel strip surface. Measurement.

[20] Zhao C., Shu X., Yan X., Zuo X., Zhu F. (2023). RDD-YOLO: A modified YOLO for detection of steel surface defects. Measurement.

[21] Liu G., Ma Q. (2023). Strip steel surface defect detecting method combined with a multi-layer attention mechanism network. Meas. Sci. Technol..

[22] Yu Y., Chan S., Tang T., Zhou X., Yao Y., Zhang H. (2023). Surface Defect Detection of Hot Rolled Steel Based on Attention Mechanism and Dilated Convolution for Industrial Robots. Electronics.

[23] Fu M., Wu J., Wang Q., Sun L., Ma Z., Zhang C., Guan W., Li W., Chen N., Wang D. (2023). Region-based fully convolutional networks with deformable convolution and attention fusion for steel surface defect detection in industrial Internet of Things. IET Signal Process..

[24] Wang X., Zhuang K. An improved YOLOX method for surface defect detection of steel strips. Proceedings of the 2023 IEEE 3rd International Conference on Power, Electronics and Computer Applications (ICPECA).

[25] Zhang Y., Wang W., Li Z., Shu S., Lang X., Zhang T., Dong J. (2023). Development of a cross-scale weighted feature fusion network for hot-rolled steel surface defect detection. Eng. Appl. Artif. Intell..

[26] Bi Z., Wu Q., Shan M., Zhong W. (2022). Segmentation-based Decision Networks for Steel Surface Defect Detection. J. Internet Technol..

[27] Tian R., Jia M. (2022). DCC-CenterNet: A rapid detection method for steel surface defects. Measurement.

[28] Li M., Wang H., Wan Z. (2022). Surface defect detection of steel strips based on improved YOLOv4. Comput. Electr. Eng..

[29] Zheng Z., Hu Y., Zhang Y., Yang H., Qiao Y., Qu Z., Huang Y. (2022). Casppnet: A chained atrous spatial pyramid pooling network for steel defect detection. Meas. Sci. Technol..

[30] Ding X., Zhang X., Zhou Y., Ding G. (2022). Scaling Up Your Kernels to 31 × 31: Revisiting Large Kernel Design in CNNs. arXiv.

[31] Liu S., Chen T., Chen X., Chen X., Xiao Q., Wu B., Pechenizkiy M., Mocanu D., Wang Z. (2022). More convnets in the 2020s: Scaling up kernels beyond 51 × 51 using sparsity. arXiv.

[32] Han Z., Jian M., Wang G.G. (2022). ConvUNeXt: An efficient convolution neural network for medical image segmentation. Knowl. Based Syst..

[33] Yu W., Zhou P., Yan S., Wang X. (2023). InceptionNeXt: When Inception Meets ConvNeXt. arXiv.

[34] Lin T.Y., Dollar P., Girshick R., He K., Hariharan B., Belongie S. Feature pyramid networks for object detection. Proceedings of the 2017 IEEE Conference on Computer Vision and Pattern Recognition(CVPR).

[35] Zhao H., Shi J., Qi X., Wang X., Jia J. Pyramid scene parsing network. Proceedings of the 2017 IEEE Conference on Computer Vision and Pattern Recognition(CVPR).

[36] Chen L.C., Papandreou G., Kokkinos I., Murphy K., Yuille A.L. (2018). DeepLab: Semantic Image Segmentation with Deep Convolutional Nets, Atrous Convolution, and Fully Connected CRFs. IEEE Trans. Pattern Anal. Mach. Intell..

[37] Zhang H., Zu K., Lu J., Zou Y., Meng D. (2021). EPSANet: An efficient pyramid split attention block on convolutional neural network. arXiv.

[38] Tang R., Liu Z., Song Y., Duan G., Tan J. (2023). Hierarchical multi-scale network for cross-scale visual defect detection. J. Intell. Manuf..

[39] Yeung C.C., Lam K.M. (2022). Efficient fused-attention model for steel surface defect detection. IEEE Trans. Instrum. Meas..

[40] Li K., Wang X., Ji L. Application of multi-scale feature fusion and deep learning in detection of steel strip surface defect. Proceedings of the 2019 International Conference on Artificial Intelligence and Advanced Manufacturing(AIAM).

[41] Guo Z., Wang C., Yang G., Huang Z., Li G. (2022). Msft-yolo: Improved yolov5 based on transformer for detecting defects of steel surface. Sensors.

[42] Jocher G., Nishimura K., Mineeva T. (2020). YOLOv5. Code Repository. https://github.com/ultralytics/yolov5.

[43] NEU Surface Defect Database. http://faculty.neu.edu.cn/songkechen/zh_CN/zdylm/263270/list/index.html.

[44] Zhang S., Chi C., Yao Y., Lei Z., Li S.Z. Bridging the gap between anchor-based and anchor-free detection via adaptive training sample selection. Proceedings of the IEEE/CVF Conference on Computer Vision and Pattern Recognition (CVPR).

[45] Liu Z., Lin Y., Cao Y., Hu H., Wei Y., Zhang Z., Lin S., Guo B. Swin transformer: Hierarchical vision transformer using shifted windows. Proceedings of the IEEE/CVF international conference on computer vision.

[46] Redmon J., Farhadi A. (2018). Yolov3: An incremental improvement. arXiv.

[47] Jocher G., Chaurasia A., Qiu J. (2023). YOLO by Ultralytics. https://github.com/ultralytics/ultralytics.

